# Developing Benign Ni/g-C_3_N_4_ Catalysts for CO_2_ Hydrogenation: Activity and Toxicity Study

**DOI:** 10.1021/acs.iecr.2c00452

**Published:** 2022-05-20

**Authors:** Izabela S. Pieta, Barbara Gieroba, Grzegorz Kalisz, Piotr Pieta, Robert Nowakowski, Mu. Naushad, Anuj Rathi, Manoj B. Gawande, Anna Sroka-Bartnicka, Radek Zboril

**Affiliations:** † Institute of Physical Chemistry Polish Academy of Science, Kasprzaka 44/52, 01-224 Warsaw, Poland; ‡ Independent Unit of Spectroscopy and Chemical Imaging, Medical University of Lublin, Chodzki 4a, 20-093 Lublin, Poland; § Department of Chemistry, College of Science, King Saud University, P.O. Box 2455, Riyadh 11451, Saudi Arabia; ∥ Chemistry Innovation Research Center, R&D, Jubilant Biosys, Knowledge Park II, Greater Noida, Uttar Pradesh 201310, India; ⊥ Regional Centre of Advanced Technologies and Materials, Czech Advanced Technology and Research Institute, Palacký University, Slechtitelu 27, 77900 Olomouc, Czech Republic; # Department of Industrial and Engineering Chemistry, Institute of Chemical Technology, Mumbai-Marathwada Campus, Jalna 431 203, India; ∇ Nanotechnology Centre, Centre of Energy and Environmental Technologies, VŠB−Technical University of Ostrava, 17 listopadu 2172/15, 708 00 Ostrava-Poruba, Czech Republic

## Abstract

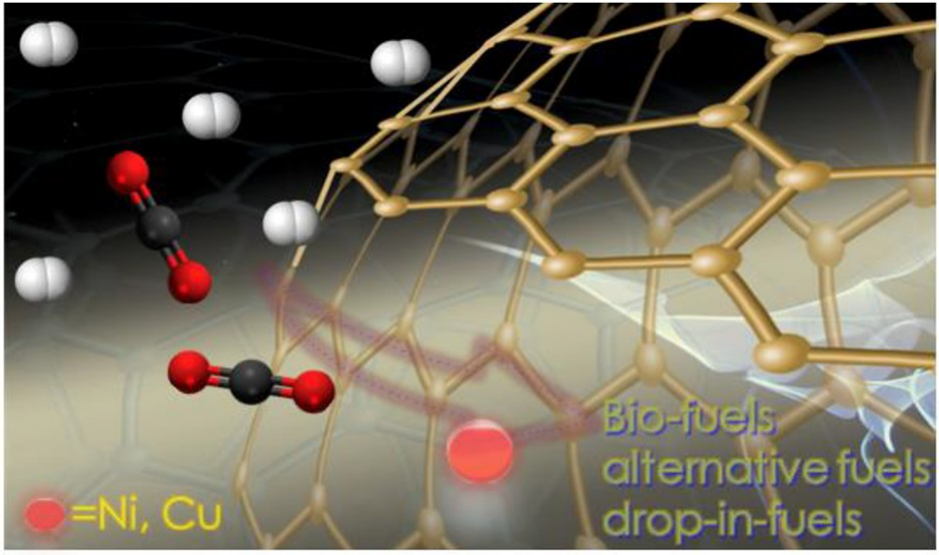

This research discusses the CO_2_ valorization via hydrogenation over the non-noble metal clusters of Ni and Cu supported on graphitic carbon nitride (g-C_3_N_4_). The Ni and Cu catalysts were characterized by conventional techniques including XRD, AFM, ATR, Raman imaging, and TPR and were tested via the hydrogenation of CO_2_ at 1 bar. The transition-metal-based catalyst designed with atom-economy principles presents stable activity and good conversions for the studied processes. At 1 bar, the rise in operating temperature during CO_2_ hydrogenation increases the CO_2_ conversion and the selectivity for CO and decreases the selectivity for methanol on Cu/CN catalysts. For the Ni/CN catalyst, the selectivity to light hydrocarbons, such as CH_4_, also increased with rising temperature. At 623 K, the conversion attained ca. 20%, with CH_4_ being the primary product of the reaction (CH_4_ yield >80%). Above 700 K, the Ni/CN activity increases, reaching almost equilibrium values, although the Ni loading in Ni/CN is lower by more than 90% compared to the reference NiREF catalyst. The presented data offer a better understanding of the effect of the transition metals’ small metal cluster and their coordination and stabilization within g-C_3_N_4_, contributing to the rational hybrid catalyst design with a less-toxic impact on the environment and health. Bare g-C_3_N_4_ is shown as a good support candidate for atom-economy-designed catalysts for hydrogenation application. In addition, cytotoxicity to the keratinocyte human HaCaT cell line revealed that low concentrations of catalysts particles (to 6.25 μg mL^–1^) did not cause degenerative changes.

## Introduction

In recent years, technologies that facilitate the creation of clean energy from renewable and sustainable resources have attracted increased attention.^1−3^ Much research and pilot work is dedicated to developing efficient, cost-effective synthetic fuel/biofuel production methods in a carbon-neutral or carbon-negative manner.^3−9^ In search of potential starting candidates for future chemical building blocks, CO_2_, one of the greenhouse gases (GHGs), has captured industrial interest because it is an abundant, inexhaustible, and inexpensive carbon feedstock.^2,10−13^ At the same time, its hydrogenation to energy-rich products can offer diverse hydrocarbon compounds such as methane, methanol, formic acid, formaldehyde, and C1–C2 ethers.^7−9,12,14,15^ Because of the variety of possible applications, including the rubber industry, pharmacy, agriculture, and food technology, they are considered to be highly attractive products. They can also be directly employed as a hydrogen-rich source/energy vector for fuel cells.^8,15−17^

CO_2_ hydrogenation is a highly exothermic process, where the reaction equilibrium is significantly influenced by pressure and temperature.^8,14,16,18^ The hydrogenation reaction research concentrates mainly on C_1_ or short-chain products CO, HCOOH, CH_3_OH, CH_4_, and C_2_–C_4_ olefins.^12,19^ For that, two kinds of mechanisms have been proposed. The first one involves CO_2_-to-CO conversion via a reverse water–gas shift (RWGS) reaction and subsequently CO methanation according to eqs 1 and 2, respectively.^2,14^
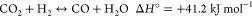
1
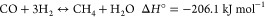
2

The second proposed mechanism considers the direct hydrogenation of CO_2_ (eq 3) via intermediate species formation, i.e., formate species.^18^
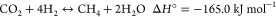
3

Materials development plays an essential role in extensive technological innovation for clean energy conversion and storage. Mostly used in the hydrogenation process, transition-metal-based catalysts are loaded with active phases and are considered to be toxic materials, with dedicated disposal protocols.^20^ Considering the vast amounts of spent catalysts worldwide, especially fluid catalytic cracking (FCC) catalysts, an alternative is needed to prevent extensive traditional landfill disposal, reduce environmental contamination, and reduce hazardous solid wastes.^21^ Current research activities focus on a new catalyst preparation methodology that applies solvent-free protocols ideally, atom economy principles, new catalyst compositions, catalyst stability, and durability. Both noble-metal-based catalysts, i.e., Pt, Rh, Ru, and Ir, and transition-metal monometallic catalysts, i.e., Ni and Ni–V bimetallic systems, were found to be effective for the CO_2_ valorization process.^8,14,17,22−25^ However, non-noble-metal small clusters or single-atom catalysts (SACs) for the hydrogenation of CO_2_ to gas or liquid products, including fuels/drop-in fuels, remain rare.^5,26,27^ For CO_2_ methanation reactions, Ni-based catalysts (usually up to 20–30% Ni content) are usually applied in real-scale industrial lines because of their good catalytic performance and for economic reasons.^14^ However, their application potential is limited, both at high and low temperatures, because of catalyst coking and Ni nanoparticle (NPs) sintering and poor activity, respectively.^14^ Recently, vanadium-modified Ni 2D nanocatalysts have been reported, delivering exceptional conversions for low-temperature hydrogenation.^8^ Moreover, the NiV catalyst, obtained from a hydrotalcite precursor, outperformed the best-known catalyst and presenting at the same time high durability and selectivity (the equilibrium conversion occurred at 623 K, and the primary product of the reaction was CH_4_ (>97% CH_4_ yield)). Copper is another transition metal considered for hydrogenation reactions both as (1) a Cu-based catalyst with a high specific surface area and semiconductor properties (usually up to 60–70% Cu content), i.e., Cu–ZrO_2_ and Cu/ZnO/ZrO_2_ for the CO_2_-to-methanol reaction, and (2) Cu complexes, i.e., copper(I) complex LCu(MeCN)PF_6_ for CO_2_ hydrogenation to formate/formic acid in the presence of DBU as the base.^28,29^ Applying higher pressure, usually 5–40 atm, supported Cu-based catalysts being used in various industrially relevant hydrogenation processes, i.e., methanol synthesis, the low-temperature water–gas shift, and various organic compounds’ selective hydrogenation or fine chemical synthesis.^30^ Here, the metal/oxide interface, the synergy between Cu^0^ sites and −OH groups, and basic Lewis sites shape the overall catalyst activity and selectivity.^31,32^ For example, in the case of Cu/ZnO/ZrO_2_ catalysts, hydrogen adsorption and dissociation are carried out by Cu sites. The basic sites of ZnO and ZrO_2_ are responsible for CO_2_ adsorption (as a mixture of carbonate and bicarbonate species). Because both sites are in close proximity to each other, the reaction is facilitated. The atomic hydrogen spills over from the Cu^0^ surface to the surface of basic sites, hydrogenating the adsorbed carbonate species to formate, methoxide species, and methanol.^33^

For the efficient adsorption and activation of CO_2_, various carriers are investigated that exhibit the desired structural properties due to oxygen vacancies and reversible valence shifts. Carbon materials, including doped carbons, graphene oxide (GO), reduced graphene oxide (rGO), and carbon nanostructures (i.e., carbon nanofibers CNFs), are also studied because of their exceptional morphological and chemical properties, adjustable surface chemistry, high thermostability, electrical conductivity, and high adsorption of hydrogen.^3,34−37^ Among those materials, graphitic carbon nitride (g-C_3_N_4_) possesses unique chemical properties and great mechanical and chemical stability. g-C_3_N_4_ sites that can serve as Brønsted acids (N–H sites) and Lewis bases (N lone electron pairs) are present, which can be further modified by alkanization or protonation.^15,38^ Besides, within this material, the catalyst nanoparticles can be anchored, and sulfur, boron, or metal particles (e.g., Fe, Cu, and Ni) can be doped to enhance the electrical conductivity as well as the photo- and electrocatalytic activity^15,38^ Pristine g-C_3_N_4_ is a semiconductor with a 2.5–2.8 eV band gap. Structurally, g-C_3_N_4_ is similar to some degree to the N-substituted graphite framework with a very high level of nitrogen doping. It comprises π-conjugated graphitic planes formed from sp^2^-hybridized carbon and nitrogen atoms.^13,15,35,37,38^ Pristine or doped g-C_3_N_4_ is tested in various applications in hydrogen evolution, the oxidation of C–H, C–C, N–H bonds, the degradation of organic pollutants, sensing, bioimaging, and gas storage.^15,38^

In this work, graphitic carbon nitride (g-C_3_N_4_) has been used to prepare a transition-metal-based catalyst (Ni, Cu, and Cu–Ni) with improved stability in CO_2_ hydrogenation. The hypothesis is that, similar to other carbon materials, g-C_3_N_4_ offers a variety of sites type and can promote the reaction by stabilizing metal clusters/nanoparticles and providing good CO_2_ adsorption and H_2_ storage. Moreover, in the current work, the Cu influence on the catalysts’ selectivity and durability in CO_2_ hydrogenation is shown. The catalysts’ performances were compared with those of commercially available best-performing reference material (NiREF).^39,40^ Their toxicities were evaluated using an *in vitro* study of keratinocyte cell lines from histopathologically normal adult human skin. Because of numerous reports on the cytotoxicity of Cu, Ni catalysts, and their oxides, we decided to estimate the influence of these particles on human skin cells (HaCaT line). The morphological changes were determined microscopically, and metabolic disorders of cells were estimated in the MTS assay over a wide range of concentrations (3.125–500 μg mL^–1^). This allowed for assessing the biological safety of the use of synthesized catalysts particles.

## Results and Discussion

### Physicochemical Characterization

**Figure 1:**
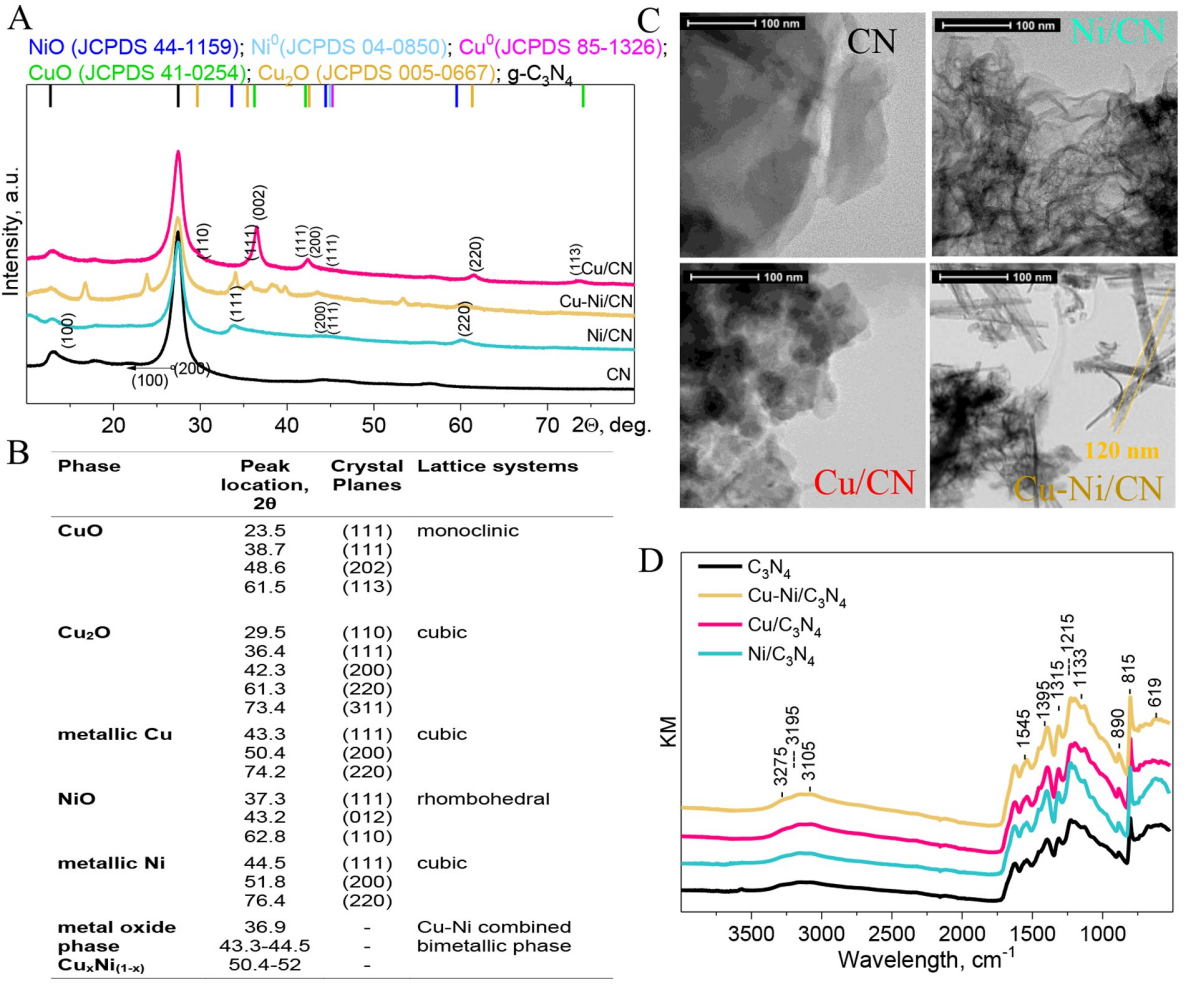
(A) XRD patterns of the calcined samples with (B) the assignment of the corresponding XRD peaks (based on SI(15)). (C) HRTEM and (D) ATR spectra of CN-supported catalysts.

The physicochemical data that apply the procedures described in the SI are presented in Table 1 and Figure 1. All homemade g-C_3_N_4_ samples were characterized by a roughly constant *S*_BET_ of ±20 m^2^g^–1^, while the *S*_BET_ of NiREF industrial full-grain samples was very low 2 m^2^ g^–1^ (specific for α-Al_2_O_3_).

**Table 1:** Physicochemical Properties Data Resume of Studied Catalysts

catalyst	description	Ni or/and Cu content, %	*S*_BET_, m^2^/g_cat_	H_2_ consumption *T*, K	*D*_pNiO_, nm^a^
Ni-V/α-Al_2_O_3_	NiREF	13.2	2.0	603, 673, >750	<12
g-C_3_N_4_	CN	0	179		
Cu/g-C_3_N_4_	Cu/CN	4	157	650, >730	<20
Cu-Ni/g-C_3_N_4_	Cu-Ni/CN	4	161	>730	<4/130
Ni/g-C_3_N_4_	Ni/CN	4	167	>730	<1

a Obtained from a XRD study.^38^

The XRD patterns of the catalysts displayed in Figure 1 are dominated by a sharp peak at 2θ = 27.4°, characteristic of graphitic materials and ascribed to π-conjugated layers stacked with index (0 0 2).^37,41^ Consistent with previous studies, the interlayer spacing of the stacked nanosheets is 0.32–0.35 nm, and the size range is 1.1–1.5 nm.^38,42,43^ Moreover, in all prepared hybrid catalysts, fine-scale nanostructures have been evidenced, as all corresponding patterns exhibit the broad features in the low 2θ region. In Figure 1B, the peaks assignment of the respective CuO, NiO, and Cu_*x*_Ni_(1–*x*)_ phases is summarized. Clear signals from the transition-metal-containing phase were observed for Cu/CN and Cu–Ni/CN catalysts, suggesting some extent of segregation and agglomeration of CuO and NiO particles, which was also confirmed by HRTEM Figure 1C. For Ni/CN, the data suggested a fine distribution of Ni/NiO over the CN support. In general, those phases were beyond the XRD detection limit. The obtained particles sizes are shown in Table 1. In all studied materials, 2D sheetlike structure with wrinkles characteristic of pure CN (Figure 1C) was observed via HRTEM. This structure was unchanged for the samples modified with Ni, Cu, or both incorporated metals (Figure 1C), in agreement with the literature.^13,15,38^

For the CN support (Figure 1D) and all investigated hybrid catalysts, five main regions were identified as being indicative of (i) −N–H stretching (3000–3200 cm^–1^), (ii) terminal C–N bonds (2100–2700 cm^–1^), (iii) aromatic conjugated C–N stretching (1400–1200 cm^–1^), and (iv) C=N stretching vibrations (1650–1540 cm^–1^) and CN heterocycle vibrations of the triazine ring (800–700 cm^–1^).^15,44,45^ The broad bands at 3000–3500 cm^–1^, most pronounced for the Cu–Ni/CN catalyst and assignable to the OH stretching mode ν(OH)^−^, were due to physisorbed water on the catalyst surface.^33,36,46^

**Figure 2:**
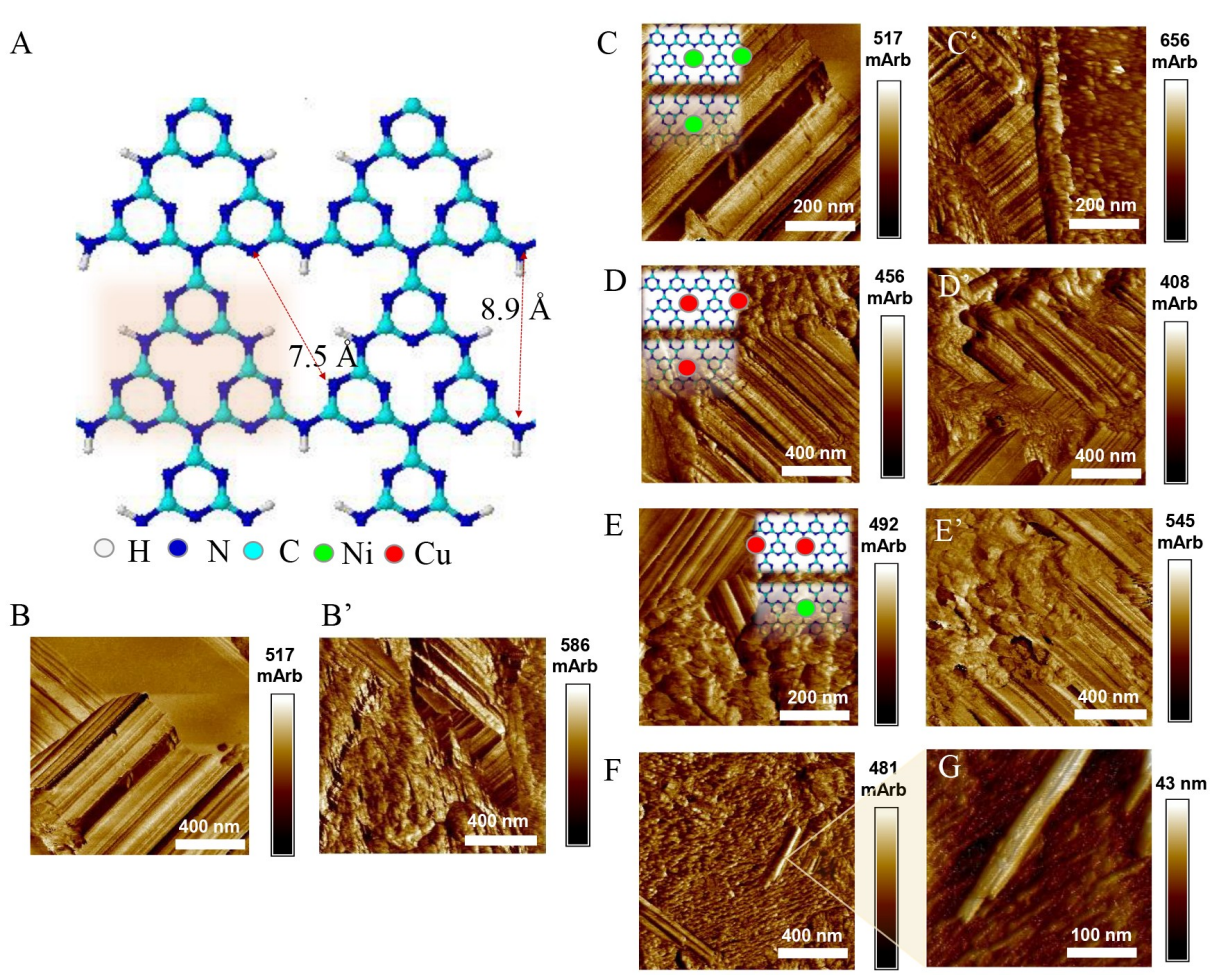
(A) Optimized structural elements of graphitic carbon nitride: heptazine (tri-*s*-triazine) units (marked) linked by bridging −NH– groups and N–H groups on their edges forming condensed heptazine rings. (B−G) AFM topography images of CN before (B) and after reaction (B′), Ni/CN before (C) and after reaction (C′), Cu/CN before (D) and after reaction (D′), and Cu−Ni/CN before (E−G) and after reaction (E′). The films were deposited on HOPG, images were acquired in air at room temperature.

Figure 2A shows the optimized structure of g-C_3_N_4_. The heptazine (tri-*s*-triazine) units are highlighted in pink in Figure 2A. They are linked by N–H groups on their edges and bridging −NH– groups to form condensed heptazine rings. In bare CN, the interplanar pores and the 6-fold cavity size stay close to 3.2 and ca. 7.3 Å, respectively.^7,28,29^ The dimension of Ni NPs was shown to be <1 nm, indicating possible Ni insertion and NP stabilization within the CN cavity, as was suggested before.^38^ A similar effect was observed for Pd and Pt atoms deposited on g-C_3_N_4_.^47^ The 6-fold cavity of g-C_3_N_4_ was estimated by DFT calculation to stabilize Pd and Pt atoms, and the binding energies were given as −2.17 and −2.95 eV, respectively. Furthermore, it was shown and evidenced by STM microscopy that, very similar in chemical structure to CN, the porphyrin cavity (i.e., comprising N, C and H), although smaller in dimensions compared to the CN cavity, was able to stabilize Ni and Co.^30^ However, within the CN cavity, atoms such as V, Cr, Mn, and Fe were shown to be more stable than their bulk phases.^7^ At the same time, preferential segregation and metal cluster formation for Co, Ni, Cu, and Zn were reported.^7^ This tendency has also been evidenced in this work by an AFM microscopy study for fresh and after-reaction catalyst samples. The AFM topography images of the graphitic nitride support deposited on the HOPG surface are displayed in Figure 2B for fresh samples and in Figure 2B′ for after-reaction samples (samples after catalytic study in the hydrogenation reaction). Graphitic nitride islands of hundreds of nanometers are evidently distinguished in the presented images. They are characterized by relatively smooth surfaces with characteristic linear steps suggesting a layer structure (with a layer thickness in the range of 5–10 nm). These typical elements are also visible for all studied samples containing Cu, Ni, and both transition metals (Figure 2C–E for Ni/CN, Cu/CN, and Cu–Ni/CN, respectively). Moreover, the investigated layers consist of another element with more undefined (cloudlike) character, which can be related to the amorphous carbon residue. The amount of this phase increases (is more visible) in the samples after reaction (Figure 2C′–E′ for Ni/CN, Cu/CN, and Cu–Ni/CN, respectively). Moreover, for the Cu–Ni/CN catalyst, segregated Cu and Cu–Ni phases in the form of nanotubes/nanowires were evidenced at sizes of hundreds of nanometers (Figure 2F,G).

**Figure 3:**
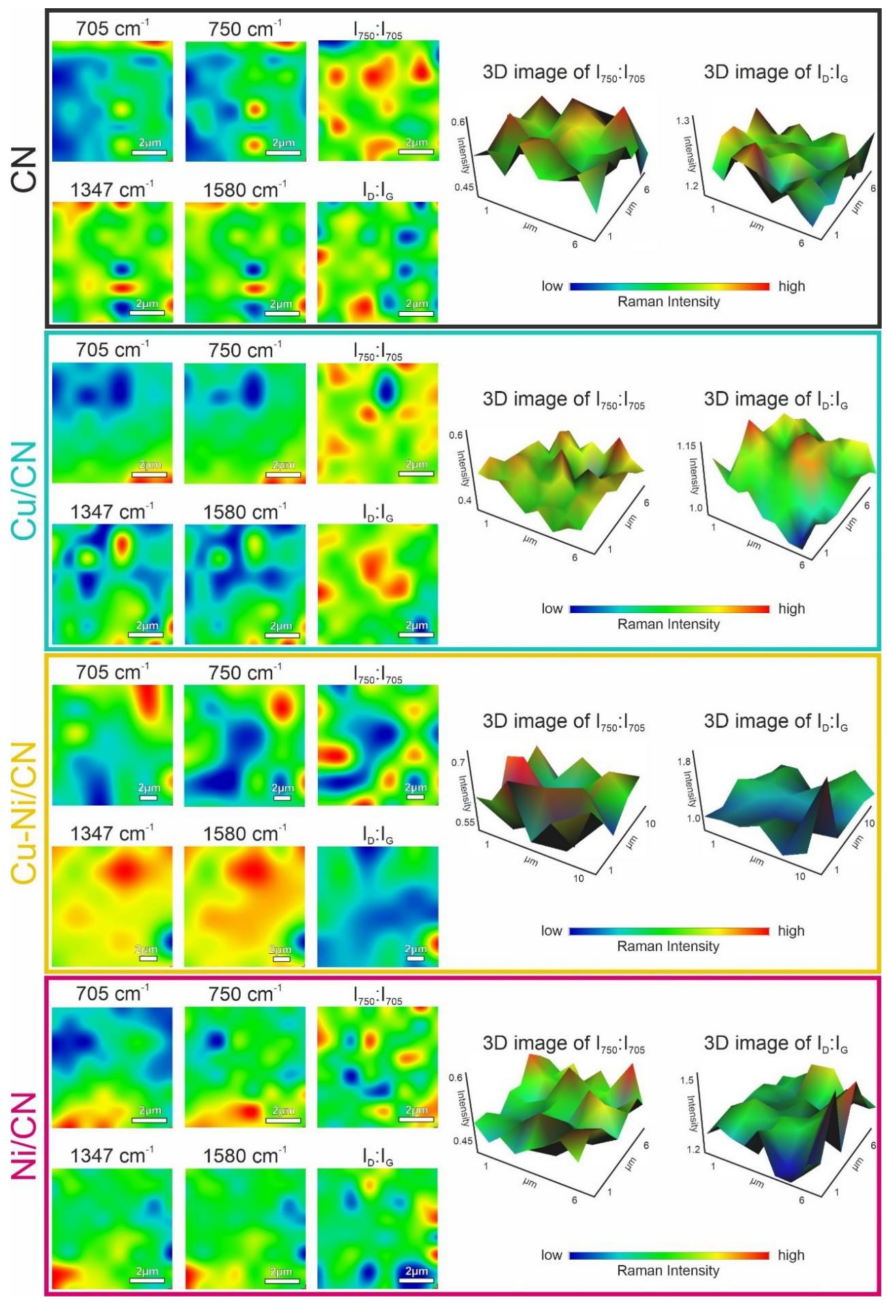
Raman images of catalyst nanoparticles with a 50 μm pinhole aperture and 50× magnification. In the ROI region, distributions of 705 and 750 cm^–1^ for the D band (1347 cm^–1^) and G band (1580 cm^–1^) were visualized with intensity ratios of extent of exfoliation (*I*_705_/*I*_750_) and level of disorder (*I*_1347_/*I*_1580_). Intensity ratios are presented with 3D images on the right side of the panel.

The chemical and structural changes in the CN sample were evaluated by Raman imaging spectroscopy, and the results are presented in Figure 3. The typical bands for sp^2^ carbon materials were assigned to G (for graphite) and D (for disordered) at ca. 1570 and 1370 cm^–1^, respectively.^5,15,38^ Using the *I*_D_/*I*_G_ peak intensity ratio, the level of disorder in the bare CN and hybrid material can be characterized as described previously.^15,16,38^ Moreover, from the *I*_750_/*I*_705_ ratio (or the *I*_543_/*I*_479_ ratio), the degree of exfoliation (extent of exfoliation) can be obtained.^38^ In all studied catalysts, the *I*_750_/*I*_705_ ratio indicated the presence of bulk g-C_3_N_4_; i.e., in the case of pure CN and hybrid Ni/CN, the *I*_750_/*I*_705_ ratios were ca. 0.2–0.5 but they increased to 0.4–0.5 for Cu/CN and Cu–Ni/CN samples.

The structural morphology with the surface samples (Figure 3) and its changes for after-reaction samples were studied by detailed Raman mapping. This allows for visualizing any chemical changes in the studied material. High scattering for bands located at 705 and 750 cm^–1^ was evidenced for all samples, which are indicative of π-conjugated graphitic planes in g-C_3_N_4_, specifically for the overall layer numbers, layer-stacking configurations, and interlayer coupling.^15^ As shown in Figure 3, the intensity of the band at 705 and 750 cm^–1^ was the same within the whole sample (described as the ratio of bulk coplanar g-h-heptazine-C_3_N_4_), confirming the homogeneous distribution of investigated surface species within gCN material. The derived maps (Figure 3), acquired with the pinhole aperture, enabled the surface samples to be characterized at a specific depth of about ca. 2 μm. These maps (Figure 3) demonstrate periodic spots, and the uniform distribution of bands confirms the interval variation of the intensity of each band and thus the homogeneity of the samples throughout the region as well as the lack of significant changes in morphology for the postreaction samples. This is consistent with the sample morphology results obtained by HRTEM and AFM analysis.

**Figure 4:**
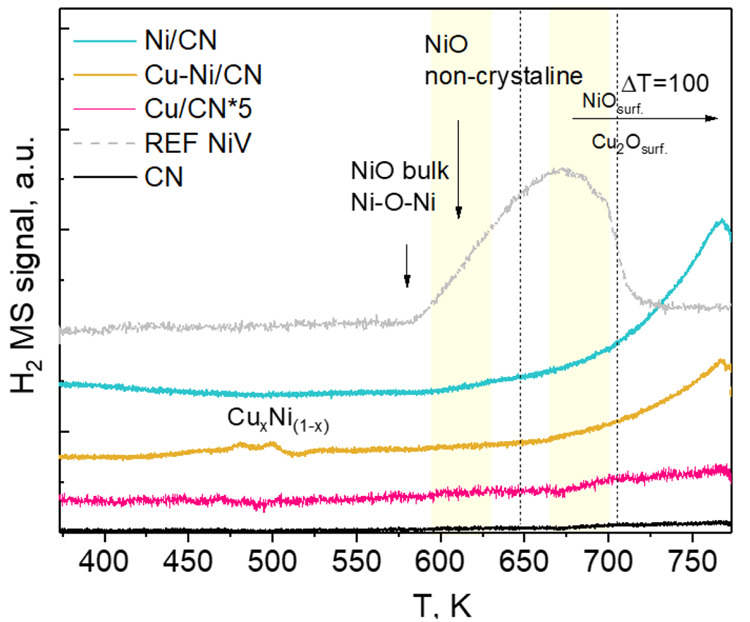
TPR profiles of the catalysts (5% H_2_ in He).

**Figure 5:**
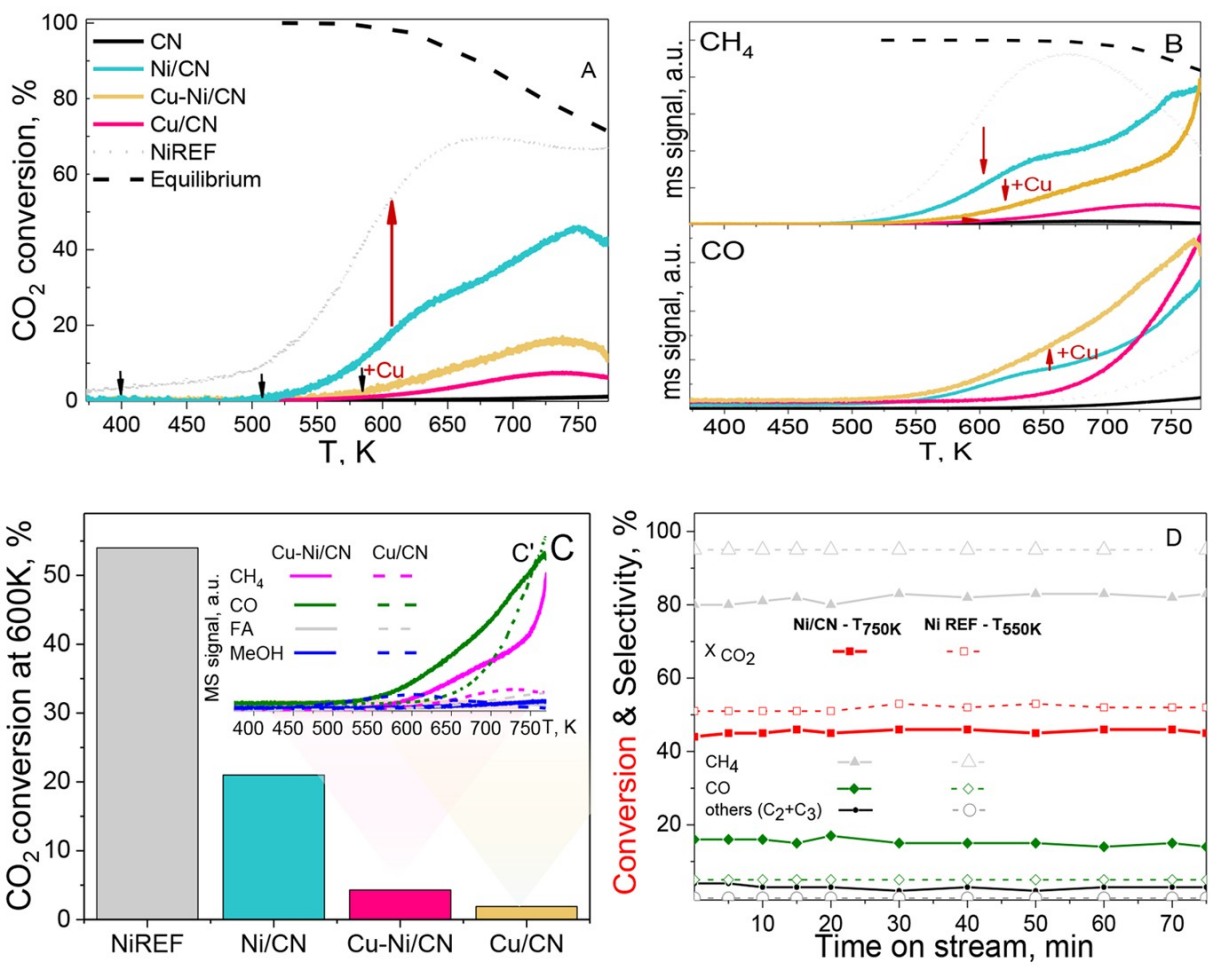
(A) CO_2_ conversion. (B) MS profiles of CH_4_ and CO. (C) Catalyst activity measured as CO_2_ conversion at 600 K with (C′) the product distribution for Cu/CN and Cu–Ni/CN. (D) Concentrations as a function of temperature and for Ni/CN- and NiREF-supported catalysts (NiREF performance given for comparison). Reaction conditions GHSV = 1.2 × 10^4^ h^–1^, 1 atm, 293 K, and CO_2_/H_2_ in a 1/5 molar ratio.

The temperature-programmed reduction (TPR) profiles for hybrid catalysts and reference samples are presented in Figure 4. The reducibility of the catalysts was examined in the temperature range of 298–773 K at a ramp rate of 10 K min^–1^. In Figure 5, the H_2_ consumptions as a function of temperature are presented. The TPR profiles demonstrated considerable differences between the CN-supported catalyst samples and the reference catalyst described elsewhere.^8,16^ It can be noticed that the catalyst support determines the TPR profiles in general, particularly the onset of the reduction temperature and the amount of hydrogen consumed. For the NiREF reference catalyst, in the TPR profile, peaks with maxima at 600, 673, and above 773 K are visible. They can be linked to the reduction of selected phases, namely, (i) NiO bulk and small NiO crystallites at ca. 600 K, (ii) noncrystalline NiO species at ca. 673 K, and (iii) NiAl_2_O_4_ above 773 K.^10,11,39^ It is recognized that the reducibility of the sample is strongly governed by metal–support interactions.^3^ Free NiO that interacts weakly with the support is usually readily reducible at lower temperatures (α-type species; low reduction temperature of 573–623 K), and the nickel species that are not fully attached to the spinel are reducible at intermediate temperatures (β-type species; intermediate temperature, 623–773 K). Nickel aluminate spinels (γ-type Ni species) undergo reduction at high temperatures, i.e., above 973 K.^16^

The observed reduction temperature for CN-supported Ni (Figure 4) differs from that reported for NiREF. This discrepancy can most probably be linked to the catalyst composition, namely, the metal–support interaction and the presence of small Ni particles <1 nm.^14,16^ The peak of H_2_ consumption approaches maxima above 773 K. This is in good agreement with the literature, where the NiO bulk and noncrystalline NiO reduction are typically observed at temperatures of ∼650 and ∼750 K, respectively.^23,48,49^ For Cu-containing samples, the peak maxima related to H_2_ consumption are less intense. For Cu/CN, maxima at 625 and ∼750 K can be distinguished, while for Cu–Ni/CN, the broad signal at ∼750 K occurred again. This can be connected to noncrystalline CuO and Cu_(*x*)_Ni_(1–*x*)_ binary phase reduction in Cu/CN and Cu–Ni/CN, respectively. The evidenced slight shift of the reduction peak for Cu/CN can be indicative of a weakening NiO–support interaction, which improves the Ni reducibility.^50^

Usually, one broad band above 523 K is observed for a Cu-containing carbon-supported catalyst, indicating Cu_*x*_O to Cu reduction. Typically, this band can be deconvolved into two α and β peaks, attributable to the highly dispersed Cu_*x*_O species reduction at a lower temperature (α-peak) and the bulk Cu_*x*_O species reduction at a higher temperature (β peak), respectively.^51^ Apparently, for the Cu/CN sample, studied, the percentage of the highly dispersed Cu_*x*_O species is significantly lower than that of the bulk species, which is consistent with microscopic and XRD studies.

### Catalyst Activity

The catalytic activity results for the CN-supported catalysts and NIREF samples reduced at 773 K are given in Figure 5. The calculated equilibrium data for the CO_2_ methanation reaction for NiREF are displayed together with the MS profiles of the reactor exit gases. Before hydrogenation, all catalyst samples were activated by in situ NiO-to-Ni reduction in a stream of H_2_. An identical procedure was used for Cu/CN for comparative purposes. Online MS was used for the continuous monitoring of relevant C species, such as CH_4_ (*m*/*z* = 16), CO (*m*/*z* = 28), CH_2_O (*m*/*z* = 30), CO_2_ (*m*/*z* = 44), and H_2_O (*m*/*z* = 18). For the studied process, the primary products were selected on the basis of the reaction outlined in eqs 1–3. These reactions were taken into account owing to their higher relevance resulting from the enthalpy values.^8,16^ For Ni/CN, CO_2_ conversion is initiated at 500–520 K, while in Cu-containing catalysts (Cu–Ni/CN and Cu/CN), on the other hand, it is shifted to temperatures higher than 550 K. It must be noted that CO_2_ conversion is kinetically constrained below 573 K. Because of that, all observed CO_2_ conversion profiles are significantly below the thermodynamic equilibrium curve (Figure 5A). At the same time, for the NiREF sample, the activity increases, reaching almost equilibrium values.

For Ni/CN and NiREF catalysts, the maxima in CO_2_ conversion and CH_4_ yield are observed between 620 and 750 K (Figure 5A,B) and remain below the thermodynamic limits. Moreover, for all CN-supported catalysts, RGWS is significant, as indicated by the respective CO profiles. According to the former study, small cubic Ni nanocrystals (nanoparticles NPS) are found to be highly selective in the hydrogenation of CO_2_ to methane, while larger Ni particles are reported to be more active in the RWGS reaction leading to CO formation.^8,14,16^ However, this is valid for the particular support, which is characterized by rather weak basicity, as in the case of the NiREF sample, where alumina is used as a support.

In the cases Cu–Ni/CN and Cu/CN, although small Cu_*x*_O, the Cu_*x*_Ni_(1–*x*)_ mixed phase, and NiO crystallites were formed, the overall activity toward methanation is low (Figure 5C). CO_2_ conversions stayed far below the values allowed by equilibrium even in the higher temperature range (Figure 5A). As shown in Figure 5A,B compared to the Ni/CN- for Cu–Ni/CN-based catalysts, an increase in temperature has led to a CH_4_ formation drop, which was accompanied by enhanced CO formation.^16^ For these catalysts, CO formation commences at 500–550 K and increases with increasing temperature, reaching a maximum above 720 K. During the CO_2_ methanation process, CO has the potential to be formed mainly via the reverse water–gas shift reaction (RWGS) (eq 1).^16^ Compared to the alumina-supported NiFER catalyst, for Ni/CN the CN support decreases the selectivity to methane. Besides, in the presence of a CN support, the RWGS reaction onset was shifted at a temperature lower by 200 K. Significant amounts of CO were detected from 600 K for all Ni/CN, Cu–Ni/CN, and Cu/CN catalysts. For these catalysts, the active phase is composed of very fine and stable nanoclusters/nanoparticles stabilized within the g-C_3_N_4_ cavity, as shown by the XRD study. The NPs size follows the order Cu (20 nm) > Cu–Ni (4, 130 nm) > Ni (ca. 1 nm). Besides, in Ni/CN and Cu/CN, Ni and Cu phases are defined by XRD as mainly Ni(111) and Cu(111), respectively. For those types of surfaces presented, the DFT study considered another hydrogenation mechanism for the difference between surface and subsurface H.^17,52^ Over an alumina-supported catalyst, the CO_2_ methanation reaction is accepted to occur through an adsorbed CO intermediate via (i) the transformation of CO_2_ to CO prior to methanation or (ii) gas-phase transformation pathways not first requiring the transformation of CO_2_ to CO on the catalyst surface,^53^ with the WGS reaction possibly occurring via (i) the formate, (ii) the redox, or (3) the carbonate mechanism.^54^ Ni(111) CO_2_ hydrogenation to a formate intermediate was found to be more favorable than hydrogenation to a carboxyl intermediate.^17,52^ Furthermore, the hydrogenation to formate proceeds through a univalent structure that is promptly transformed to a bivalent structure.^17,52^ The formation of the formate species (HCOO^–^) involves at least three bond rearrangements, namely, (i) the cleavage of the O–H bond followed by (ii) the formation of the O–C and (iii) C–H bonds.^55^ The once-formed HCOO^–^ is relatively stable because its subhydrogenation to formic acid (FA) is energetically not as favorable as the formation of formate. Similar monovalent formate has been shown to be stable on Cu(111).^32,56^ Despite these species being difficult to further hydrogenate to FA, they undergo almost spontaneous transformation to bidentate formate, as the energetic barrier for this reaction was reported ca. 0.06 eV.^17,52^ Nevertheless, the presence of formate species is suggested to decrease the number of available sites for H_2_ dissociation as well as partially/completely block the path for hydrogen surface transport.^57^ The formate coverage decreases with increasing temperature because of formate decomposition.^57,58^ Accordingly, two different pathways have been proposed for that: (i) dehydrogenation producing CO_2_ and H_2_ and (ii) dehydration where CO and H_2_O are formed.^12^ It was suggested that CO is usually released during dehydration while terminal OH (OH groups) are restored.^59^ Our activity profiles over a CN-supported catalyst (Figure 5A,B) suggest that a similar mechanism can govern the CO_2_ hydrogenation because the dehydroxylation process is observed in a similar temperature range, also with CO present in the gas phase.^55^

The comparison of catalyst activity at 600 K is shown in Figure 5C. A relatively high activity of NiREF catalysts was related to the support basicity and its CO_2_ adsorption ability.^14,16^ The Cu/CN catalyst has the lowest activity in CO_2_ hydrogenation among the CN-supported samples studied. For Cu-containing catalysts, an increase in CO selectivity was observed with traces of methanol and FA species (Figure 5C′). Cu is considered to be a relatively efficient catalyst for selective C–O bond hydrogenolysis and an excellent WGS catalyst, facilitating the conversion of CO to CO_2_ and vice versa.^32,56^ It is also reported as an efficient methanol synthesis catalyst, using both CO_2_ and/or CO as a carbon source, even at atmospheric pressure.^33^

Although the CN catalysts’ activity is much lower than that noted for NiREF (Figure 5C), i.e., with a 40% drop in CO_2_ conversion for CN-supported material, the Ni content is far less in all studied CN-supported catalysts. In comparison to NiREF, Ni content is 70% lower for Ni/CN, Cu–Ni/CN, and Cu/CN catalysts. Besides, compared to standard commercial hydrogenation catalysts, the content of Ni is lowered by almost 90%, but still the CO_2_ conversion is maintained at ca. 20% at 600 K.^14,16^ This, on the contrary, would suggest a much higher atom economy efficiency for CN-supported catalysts. This issue could also most likely be connected to the ease of hydrogen diffusion and access to the Ni or Cu sites, as was mentioned earlier for the 2D g-C_3_N_4_ structure with well-described cavities, porous structure, and interlayer distances.^13,15,19,27,30,38^ Similarly, graphitic carbon nitride nanotubes were considered to be a promising hydrogen sorbent because of their highly porous structure and doubly bound nitrogen at the edges of the pores.^60,61^ These reasons may be responsible for facilitating hydrogen diffusion into the interior of the nanotubes, thus providing active sites for hydrogen adsorption and/or functionalization with metal catalysts.

The stability test for Ni/CN was carried out at 623 K over 72 h, and the results were compared with NiREF (Figure 5D). Ni/CN catalysts exhibit a CO_2_ conversion barely reaching 50%, with CH_4_ selectivity declining from 43.0 to ca. 41.0% over the whole on-stream test (Figure 5D). The catalysts were characterized after the 72 h on-stream reaction. An AFM study showed no relevant changes in support morphology (Figure 2B′). More amorphous material was detected in the samples after reaction (Figure 2C′,D′), suggesting some extent of coking. However, no agglomeration of active phase/crystallites was observed.

Generally, nickel-based catalysts show high activity and selectivity in the CO_2_ methanation reaction, but they are easily sintered under the reaction conditions. On the other hand, Cu-based catalysts are found to be efficient for the hydrogenation of CO_2_ to hydrocarbons.^62^ Regardless of the type of catalyst used, at low CO_2_ conversions, high methanol selectivity is reported, according to eq 4. The methanol synthesis described by eq 4 is competitive with RWGS (eq 1), and the increase in reaction temperature favors the endothermic RWGS reaction.
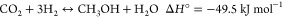
4A previous study shows that the Cu facets exposed to the reaction determine the dominant path of CO_2_ methanation. On the pristine Cu(100) surface, the CO_2_ adsorption was found to be thermodynamically unstable, yet the Cu(100) surface for the adsorption of CO_2_ molecules is reported to be more favorable than the Cu(111) surface.^63^ Besides, the preadsorbed H atoms are accessible at each step of CO_2_ hydrogenation, as H_2_ molecule dissociation proceeds easily over Cu under the reaction conditions. According to the DFT study, a low activation barrier was predicted for thermocatalytic CO_2_-to-methanol reduction on small Cu nanoclusters. Introducing a second metal, i.e., Zn, Ni, or Co, onto the Cu surface can enhance the catalytic activity. Here, a promoting effect was demonstrated for Zn doped on the Cu surface for Zn coverage below 0.2 mL. The increased yield of methanol production was also observed for Ni/Cu(100) and Co/Cu(100) surfaces as well as for Rh-, Pt-, Pb-, and Au-substituted Cu atoms on the top layer of the Cu(111) surfaces. For Cu–Zn–Zr catalysts, it has been demonstrated that the CO_2_–adsorbent interaction can be weakened by Cu–O bond formation.^63^ Therefore, the low conversion rates and CO_2_ reduction selectivities appear to be due to the thermodynamically unstable adsorption of crucial intermediates such as H_2_CO and H_2_COO on the clean Cu surface. Besides, both Cu^+^ and Cu^0^ species were reported to be essential for methanol selectivity, while the Cu^+^/Cu^0^ ratio determines the specific activity.^64^ The second metal that is doped can inhibit or promote the desired reaction pathway. For example, Zn in the case of the Cu/ZnO-based catalyst was shown to have a considerable promoting effect in methanol synthesis, whereas RWGS was not influenced. In the case of CN-supported catalysts for all systems, the formation of FA or methanol stayed below 5 ppm, and Ni addition to Cu enhanced the CO_2_ methanation and increased the amount of CO formed at a higher temperature likely via RWGS. This could also suggest that methane is mainly formed from CO_2_ rather than CO hydrogenation.

### Catalyst Toxicity Study

#### Influence of Catalysts on the Morphology of HaCaT Cells

**Figure 6:**
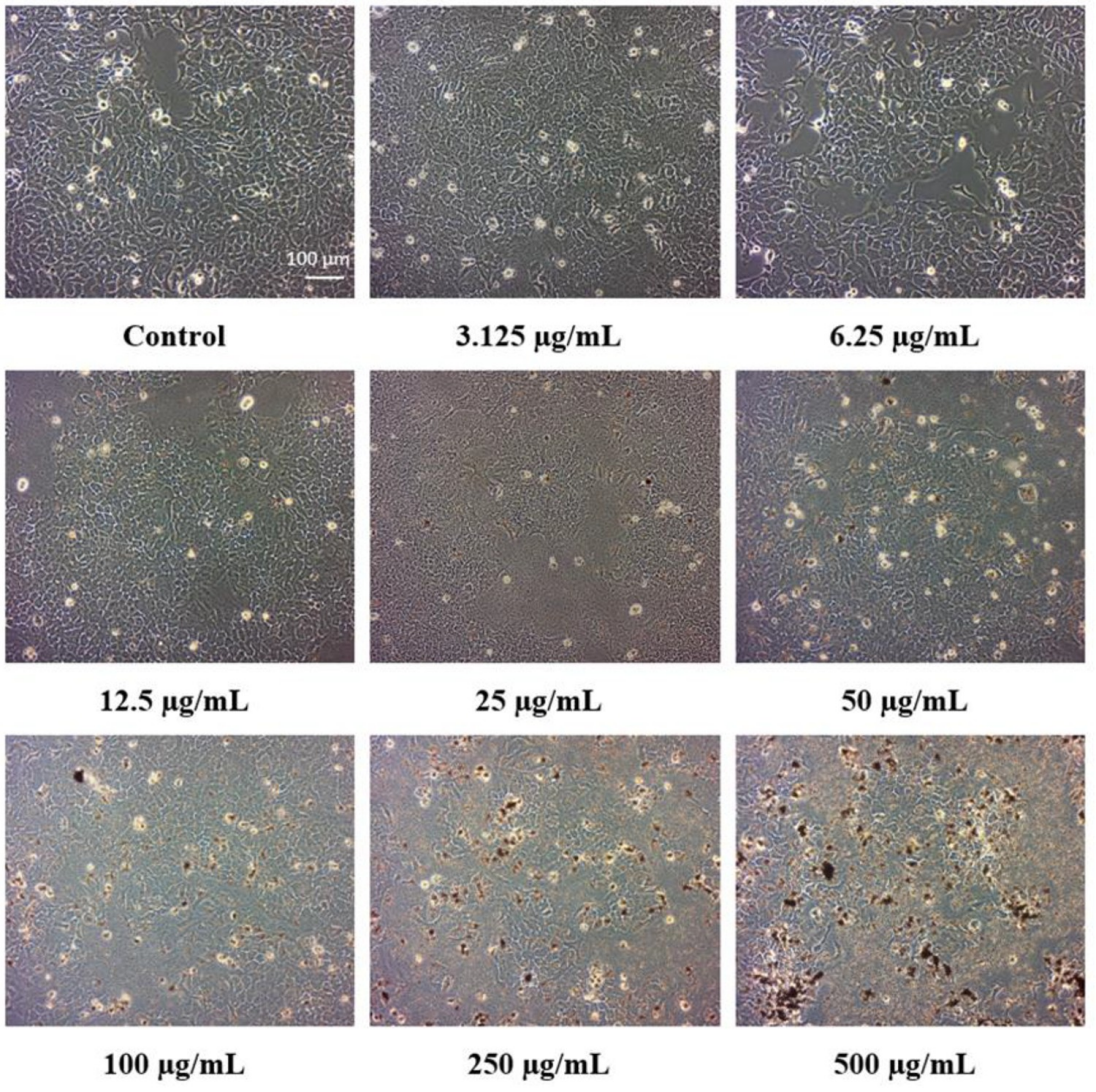
Determination of HaCaT cell morphology. The cells were incubated for 24 h alone or treated in the presence of increasing concentrations of nanoparticles. The white scale bar corresponds to 100 μm.

**Figure 7:**
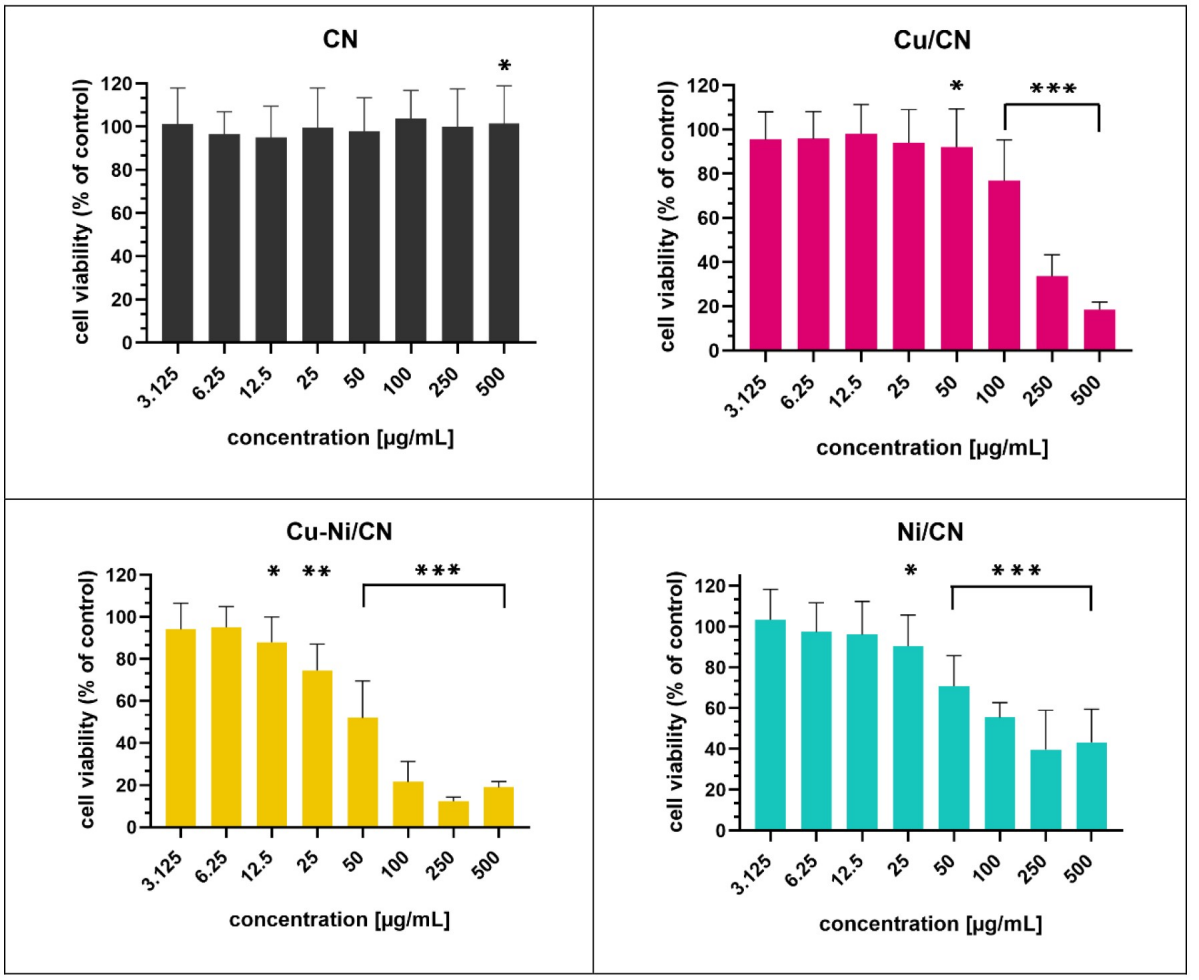
HaCaT cell viability determined using the MTS assay after 24 h of coculture with increasing concentrations of catalyst particle solutions compared to control cells cultured in SFM. *p < 0.05, **p < 0.01, and ***p < 0.001 in comparison to the control.

**Figure 8:**
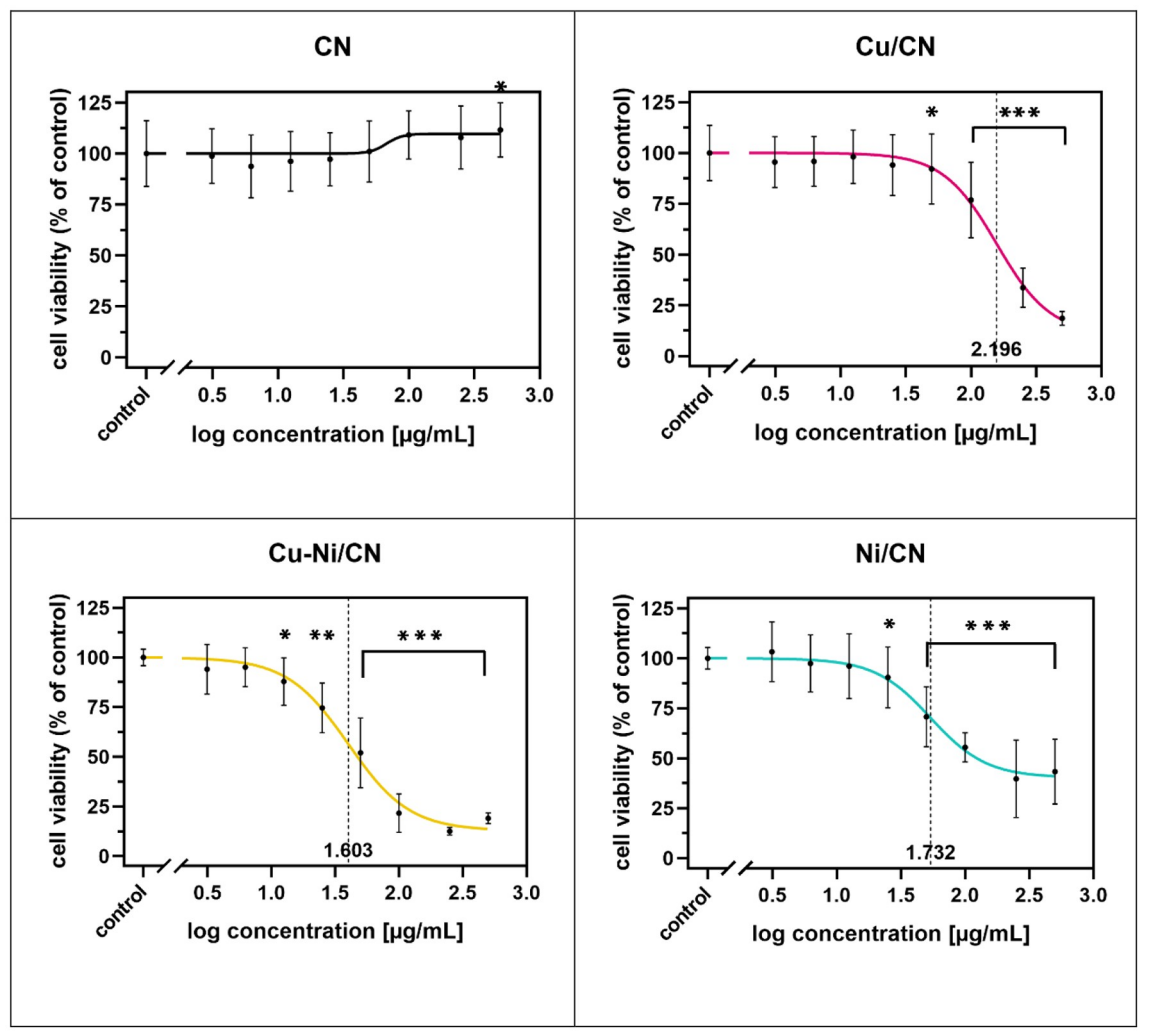
Influence of catalysts on HaCaT cell viability/proliferation (log dose–response curves). The data are expressed as the mean ± SD, *n* = 18. The log IC50 values are marked with a dashed line. *p < 0.05, **p < 0.01, and ***p < 0.001 in comparison to the control.

**Figure 9:**
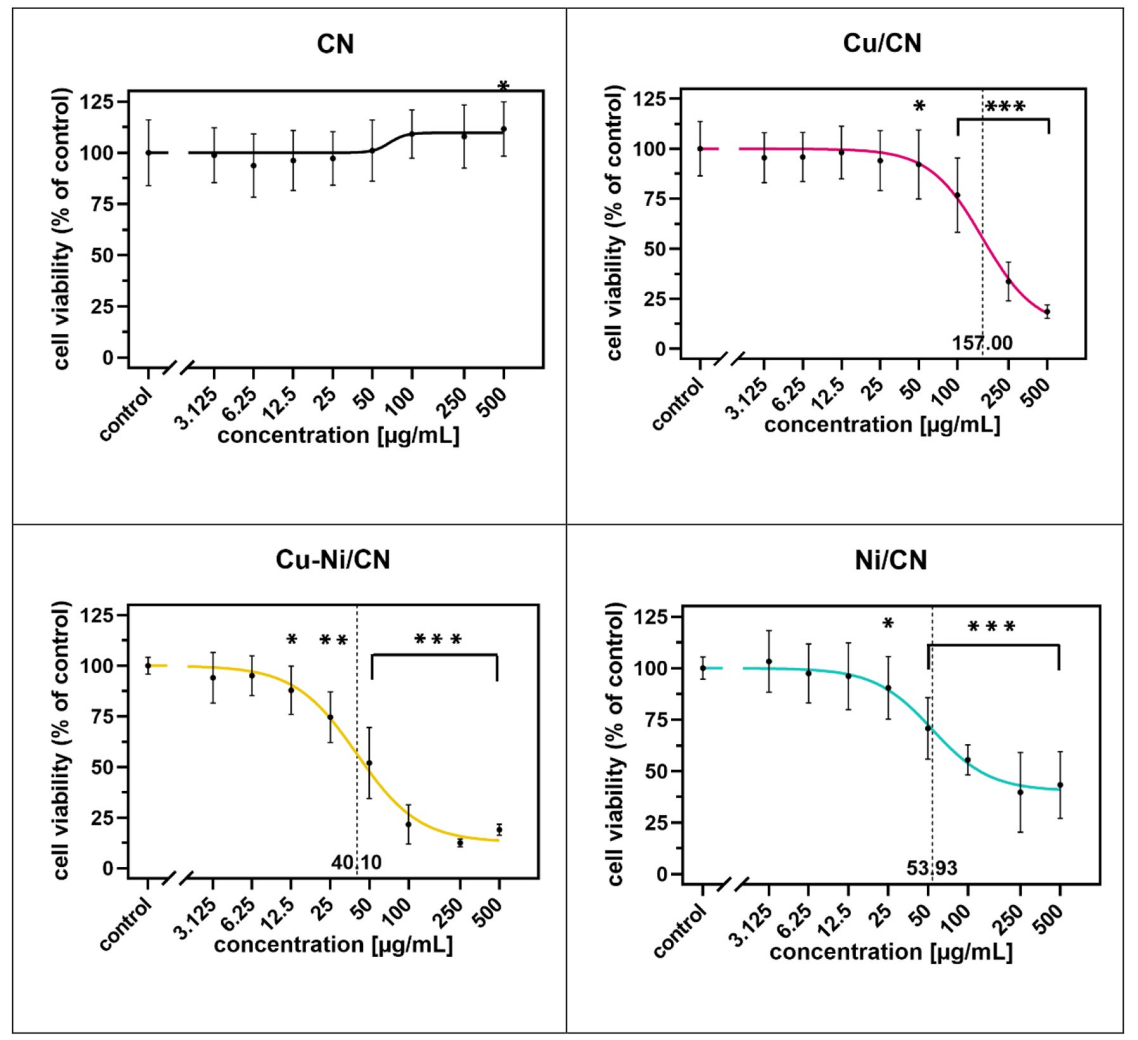
Influence of catalysts on HaCaT cell viability/proliferation (dose–response curves). The data are expressed as the mean ± SD, *n* = 18. The IC50 values are marked with a dashed line. *p < 0.05, **p < 0.01, and ***p < 0.001 in comparison to the control.

The microscopic images showed that the HaCaT cells incubated for 24 h with catalyst samples demonstrated dose-dependent alterations in the morphology and that at the highest concentrations (250 and 500 μg mL^–1^) there was a reduction in their number (Figure 6). In contrast, the untreated control cells were characterized by morphology that was normal and typical for this cell line and a proper high density (Figures 7–9). Cells treated with a low concentration of studied catalysts particles (3.125–25 μg mL^–1^) elicited only minor morphological changes compared to the control cells, which showed only a reduced number of cell-division-dependent spherical floating cells. At higher concentrations, HaCaT cells also demonstrated medium deviations in size, shape, volume, and structure, and cytoplasmic vacuolization occurred (Figure 6), indicating a moderate cellular degeneration process. Some cells are swelled and even lysed; thus, catalysts could induce the permeability disorders of the cell membrane. The precipitation of catalyst particles in the form of aggregates (dark spots) is also visible. These morphological abnormalities were the most pronounced at a 100 μg mL^–1^ concentration, but they can also be seen at 25 μg mL^–1^. The results (images) are presented for the Cu/CN sample, but other samples caused similar changes, except the CN sample, where no symptoms of cell degeneration were observed.

#### Impact of Catalysts on the Viability/Proliferation of HaCaT Cells

The viability of the HaCaT cells treated with catalysts was determined with the MTS assay after 24 h of incubation (Figure 7). The HaCaT cells cultured by the addition of catalysts particles (Cu/CN, Cu–Ni/CN, and Ni/CN samples) in the 3.125–500 μg mL^–1^ concentration range showing a dose-dependent proliferation decrease in comparison to the control, which constitutes untreated cells. They contrasted with cells subjected to the vehicle (CN sample), which did not induce a cytotoxic effect. A statistically significant effect (*p* < 0.05) was observed at concentrations of 50 μg mL^–1^ in the Cu/CN sample, 12.5 μg mL^–1^ in the Cu–Ni sample, and 25 μg mL^–1^ in the Ni/CN sample.

Interestingly, a statistically significant increase in cell proliferation was reported in the case of treatment with the CN sample at the highest tested concentration (500 μg mL^–1^). Incubation with 100 and 250 μg mL^–1^ Cu/CN, Cu–Ni/CN, and Ni/CN resulted in a decrease in the number of viable/proliferating HaCaT cells by 76.82–33.63, 21.56–12.47, and 55.48–39.70%, respectively. A 50% reduction (IC50) in HaCaT cell viability was observed at catalyst particle concentrations of 157.00 μg mL^–1^ (log IC50 = 2.196), 40.10 μg mL^–1^ (log IC50 = 1.603), and 53.93 μg mL^–1^ (log IC50 = 1.732) in Cu/CN, Cu–Ni/CN, and Ni/CN samples, respectively. IC50 values were calculated on the basis of the log dose–response (Figure 8) and dose–response curves (Figure 9). In the CN sample, the determination of IC50 was impossible because of the lack of 50% growth inhibition. The most pronounced cytotoxicity of catalysts was observed at doses from 100 to 500 μg mL^–1^ in tested samples. The HaCaT cells were the most sensitive to the Cu–Ni/CN treatment.

Contact with catalyst particles may occur through inhalation, ingestion, and dermal contact,^65,66^ and for this reason, we have chosen the HaCaT human keratinocyte cell line derived from normal skin. Ni nanoparticles (NPs) are the most frequently studied among the metallic NPs in relation to cell culture.^67^

Previously, it was found that Ni/NPs induced cytotoxicity in a dose-dependent manner in the 10–100 μg mL^–1^ concentration range in human breast carcinoma MCF-7 cells.^68^ The Ni/NPs caused mostly cyto-genotoxicity and oxidative stress, manifested by ROS production and GSH depletion.^68^ It was also demonstrated that nickel oxide nanoparticles (NiO NPs) at levels above 10 μg mL^–1^ exhibited cytotoxicity in human liver cancer HepG2 cells via an apoptotic pathway and reactive oxygen species (ROS) generation.^69^ For Ni/NPs, a concentration of approximately 30 μg mL^–1^ evoked a concentration-dependent morphology alteration and a cytotoxic effect after 24 h of treatment with bluegill sunfish BF-2 cells.^70^ Various abnormalities have been reported there, including lysosomal, mitochondrial, and lactate dehydrogenase activity and oxidative stress manifested by the escalation in the peroxidation of lipids (LPO), protein carbonyl (PC), glutathione sulfotransferase (GST), and glutathione peroxidase (GPX). However, the catalase (CAT) and total glutathione content (TGSH) were dose-dependent and diminished when the dose decreased.^70^ Pietruska et al. proved that Ni-containing NPs are cytotoxic to human lung carcinoma H460 and human bronchial epithelial NHBE cells at doses of up to 20 μg cm^2^ by activating the HIF-1α pathway and inducing hypoxia.^71^ Interestingly, metallic Ni nanoparticles showed less toxicity than NiO NPs, whereas Ni microparticles were nontoxic toward these cells. Furthermore, the cytotoxicity evoked by all Ni species was associated with the apoptotic response activation, which can suggest substantial carcinogenic potential due to the dose- and time-dependent caspases and polypolymerase (ADP-ribose) cleavage.^71^

Nevertheless, Ni NPs at concentrations of as low as 2 μg mL^–1^ induce the occurrence of the cytotoxic effect (oxidative stress and apoptosis activation) in human lung epithelial A549 cells.^68,69^ It has been reported that in normal human immortalized bronchial epithelial HBEC3-kt cells Ni and NiO NPs at a concentration above 10 μg mL^–1^ caused a release of inflammatory cytokines from exposed macrophages and inflammation-driven genotoxicity as a consequence.^72^ Ni NPs have been shown to enhance platelet-derived growth factor (PDGF) activity in modulating the production of chemokines in average rat pleural mesothelial 2 NRM2 cells via a mechanism that includes ROS generation and prolonged activation of protein kinase ERK-1,2.^73^ Ni NPs caused high toxicity starting at concentrations of 1–10 μM in human coronary artery endothelial cells (hCEC) and human coronary artery smooth muscle cells (hCASMC), disturbing their metabolic activity.^74^ In the human cervix epithelioid carcinoma cell line (HeLa), NiO NPs caused 20% of cells apoptosis.^75^ The cytotoxicity was determined in the concentration range of 50–200 μg mL^–1^ for 16 h, whereas the time was shortened by up to 2–6 h for the highest (i.e., 400–500 μg mL^–1^) concentration. A 400 μg mL^–1^ concentration of NiO NPs caused the lysis of the cell membrane and cell detachment from the culture plate surface due to the induction of apoptosis and necrosis.^75^ In leukemia, K562-cell-functionalized Ni NPs with positively charged groups enhanced the permeability of the cell membrane, and besides causing apoptosis, they also induced necrosis.^76^ In human fibroblasts, WI-38 cells demonstrated that nickel cylindrical nanostructures nanowires (Ni NWs) induced endoplasmic reticulum (ER) stress and, as a result, ER swelling in concentrations ≥2.25 μg mL^–1^ after 72 h of incubation.^77^

On the other hand, Cu NPs induce mutagenic changes and cause a significant increase in the number of binucleated cells with micronuclei.^77^ This can be indicative of a genotoxic risk associated with Cu/NPs exposure because bare Cu/NPs have the potential to promote DNA strand breaks and cause oxidative DNA damage. The *in vitro* studies suggest that Cu/NPs can decrease cell viability, leading to cell death. Thus, the Cu/NPs’ possible neurotoxic potential and neurodegenerative activity were demonstrated. This effect has been related to dopamine depletion, the alteration of dopaminergic gene system expression, and oxidative stress in the neurons of rats. Apart from that, Cu/NPs exert toxicological effects on the liver, kidney, and spleen in mice.^77^ However, the IC50 dose for Cu/NPs against ca. 1.71 μg mL^–1^ has been reported for human skin cancer cell A-375. Cu/NPs reduced the cell membrane rigidity, causing a genotoxic effect via DNA degradation and chromosomal condensation. They were found to induce cell cycle arrest in the G2/M phase, leading to depolarization of the mitochondrial membrane and finally to cells apoptosis.^78^ A similar action model was observed in CuO NPs toward TIC-enriched PANC1 human pancreatic cancer cell cultures. The IC50 value was 10 μg mL^–1^.^79^ A549 lung adenocarcinoma cells were found to be considerably more sensitive to the cytotoxic effects of CuO/NPs than HBEC human bronchial epithelial cells.^80^ The *in vitro* toxic potential of increasing concentrations of the Cu/NPs obtained using green chemistry (1–500 μg mL^–1^) in the proliferation and morphological characteristics of the human HepG2 cancer cell line was estimated to be 54.5%.^81^ However, another study over surface-modified Cu/NPs with broccoli green extract did not exhibit cytotoxicity in the 0.5–1.5 μM concentration range for the prostate PC-3 cancer cell lines.^82^

Bimetallic Cu–Ni NPs possess antibacterial activity and could be used in dental materials, although there are some reports that monometallic Cu/NPs also exhibit high antibacterial potency.^83−85^

The data obtained for the CN-containing material are consistent with literature data because reduced viability and morphological changes in HaCaT cells were observed from concentrations of 12.5, 25, and 50 μg mL^–1^ for Cu–Ni/CN, Ni/CN, and Cu/CN NPs samples, respectively. Indeed, in most studies that evaluated the cytotoxicity of Ni NPs or NiO NPs, cells from mammals, especially cells derived from human lungs, have been involved. This is because Ni is well recognized to cause several pulmonary diseases, including fibrosis and lung cancer.^86,87^ It is worth remembering that different cells have different cell viability under comparable conditions when exposed to Ni and Cu/NPs, but the mode of NP action seems to be similar. NP-mediated toxicity includes oxidative stress, inflammation, genetic damage, and the inhibition of cell division, consequently leading to cell degeneration and apoptosis.^77,88^ Generally, Cu/CN NPs were proven to be highly cytotoxic compared to other metal NPs.^88^ The next step should be to trace the mode of action of Ni and Cu/CN NPs in HaCaT cells and to determine whether in the case of this cell line NPs induce the same effects.

By exerting cytotoxicity on cancer cells, they exhibit great potential in oncology clinical and (bio)medical applications; they may constitute promising drug carriers by enhancing the proper cellular uptake.

## Conclusions

The technology needed to valorize CO_2_ via hydrogenation has been identified as a possible pathway to transform one GHG gas into value-added products such as chemicals, fuel feedstocks, and drop-in fuel. This makes CO_2_ a likely alternative in climate change mitigation. Although the CO_2_–methanol process is recognized as a reasonably well-established and mature approach, a vital concern for increasing the process efficiency is to identify a durable and nontoxic catalyst that ideally demonstrates high low-temperature activity.

The results presented by 2D graphitic carbon nitride-supported catalysts revealed a stable performance. All studied hybrid catalysts with active phase loading of ∼4 (wt %) were active in the CO_2_ hydrogenation reaction. Although the Ni loading in Ni/CN is lower by more than 90%, compared to the reference NiREF catalyst a conversion of close to 20% was achieved at ∼623 K, with CH_4_ being the primary reaction product (>80% CH_4_ yield).

Moreover, considering the toxicological profiles, the hybrid catalysts provide a direction for catalyst design with less toxicity to the environment and health. Twenty-four hours of treatment with Cu/CN, Cu–Ni/CN, and Ni/CN reduced the viability of HaCaT keratinocytes cells, causing degenerative morphological changes. Only CN bare g-C_3_N_4_ did not disturb the appropriate cell structure but positively affected their proliferation, even at the highest studied concentrations. Surprisingly, it shows great potential for clinical and (bio)medical applications in the fields of oncology and pharmacy as a drug carrier. All samples were nontoxic toward HaCaT cells at a concentrations of 6.25 μg mL^–1^. The highest toxicity was found for the Cu–Ni/CN sample, which caused a decrease in the viability of cells at a concentration of 12.5 μg mL^–1^. At the same time, the least toxic was the Cu/CN sample, where the antiproliferative effect was present at a 50 μg mL^–1^ concentration.

## Supporting Information Available

Experimental section and testing protocols (PDF)
