# Supplementary material for: Developing Benign Ni/g-C3N4 Catalysts for CO2 Hydrogenation: Activity and Toxicity Study
Source: Ind Eng Chem Res. 2022 May 20;61(29):10496–510. doi: 10.1021/acs.iecr.2c00452 (PMC9344432; doi:10.1021/acs.iecr.2c00452)
Supplement: Supplementary file 1 — ie2c00452_si_001.pdf [file ie2c00452_si_001.pdf]

Developing benign Ni/g-C_3N_4 catalysts for CO_2 hydrogenation – activity and toxicity study

Izabela S.Pieta [a], Barbara Gieroba [b], Grzegorz Kalisz [b], Piotr Pieta [a], Robert Nowakowski [a], Mu. Naushad [c], Anuj Rathi [d], Manoj B.Gawande [e,f], Anna Sroka-Bartnicka [b], Radek Zboril [e,g]*

[a]Institute of Physical Chemistry Polish Academy of Science, Kasprzaka 44/52, 01-224 Warsaw, Poland

[b] Independent Unit of Spectroscopy and Chemical Imaging, Medical University of Lublin, Chodzki 4a, 20-093 Lublin, Poland;

[c] Department of Chemistry, College of Science, King Saud University, P.O. Box 2455, Riyadh, 11451, Saudi Arabia;

[d] Chemistry Innovation Research Center, R&D, Jubilant Biosys, Knowledge Park II, Greater Noida, Uttar Pradesh 201310, India

[e] Regional Centre of Advanced Technologies and Materials, Czech Advanced Technology and Research Institute, Slechtitelu 27, 77900, Palacký University, Olomouc, Czech Republic;

[f] Department of Industrial and Engineering Chemistry, Institute of Chemical Technology, Mumbai-Marathwada Campus, Jalna, Maharashtra, India;

[g] Nanotechnology Centre, Centre of Energy and Environmental Technologies, VŠB–Technical University of Ostrava, 17. listopadu 2172/15, 708 00 Ostrava-Poruba, Czech Republic;

**Corresponding author. ipieta@ichf.edu.pl*

Experimental

2.1. Synthesis, materials, and reagents

The hybrid Ni- and Cu-based nanocatalysts, listed in Table 1, supported on two-dimensional (2D) carbon nitride g-C_3N_4 nanosheets were synthesized and evaluated in this work. Two-dimensional (2D) carbon nitride g-C_3N_4 nanosheets were synthesized by thermal polymerization as described previously.

The X/g-C_3N_4 (X=Ni, Cu, Cu-Ni) nanocomposites were obtained by a co-precipitation method [1]. The metal loading in these systems is expressed in (wt. %). Typically, 0.5 g g-C_3N_4 was dispersed in 175 mL water and stirred for 10 min. Then an aqueous solution of $Ni(NO_3)_2 \cdot 6H_2O$ (248 mg in 20 mL water), or $Cu(NO_3)_2 \cdot 3H_2O$ (200 mg in 20 mL water), or both solutions were added, and the reaction mixture was stirred for 18 h at room temperature (RT). After that, 1 g of sodium borohydride was added under constant stirring for another 16 h at 50 °C. Finally, the slurry was cooled to RT, and the obtained powder was collected by centrifugation, washed with methanol (30 mL x 4), and dried under reduced pressure at 60 °C.

The Ni-0.5V/Al_2O_3 (denoted as NiREF) catalysts were used as reference samples [2, 3]. The NiR catalyst was prepared by a two-stage wet impregnation method with subsequent thermal treatment. The calcium-modified alumina was used as a support for the active phase (S_{BET}= 2 m^2g^{-1}). The NiO content in the precatalyst eas ca. 17 wt. %. The nominal V content in catalyst was 0.5 wt. % (V_2O_5 content of 0.88 wt. %).

The catalysts unloaded from the experimental reactor after activity tests (spent catalysts) were additionally labeled with "AR."

2.2. Physicochemical characterization

The specific surface area (S_{BET}) was measured with the Micromeritics ASAP apparatus, using the BET method and N_2 as the adsorbate. Before the measurement, the samples were degassed at 473 K for 3h. S_{BET} was calculated on the basis of N_2 adsorption isotherms at 77 K.

X-ray diffraction patterns (XRD) were collected using a D5000 powder diffractometer (Bruker AXS) equipped with a LynxEye strip detector. Cu-Kα radiation was used with an X-ray tube operating at 40 kV and 40 mA. All measurements were performed in the Bragg–Brentano geometry.

ATR spectra were collected in the range 700-4000 cm^{-1} on a Nicolet Nexus instrument. Typically, 100 scans were collected at a resolution of 1 cm^{-1}.

The Raman data were collected using a confocal Thermo DXR Raman Microscope with a 50× air objective. The laser wavelength used was 780 nm. The parameters were optimized to obtain the best signal-to-noise ratio. The aperture was set to a 25 μm slit, and the laser power was 2.5 mW. The exposure time was 7s, and the number of exposures for one spectrum was 30. The chemical maps were made with a step size of 1μm on the x and y-axis. The entire map area was 8x8μm. The chemical map analysis was achieved in Omnic Software (v. 8.2, Thermo Fischer Scientific Inc., USA), and all maps were normalized before the examination.

HyperChem software package was used to obtain the optimized graphitic carbon nitride structures models. The internuclear distances were taken into account when determining the cavities dimensions; however, for the van der Waals size and geometry of the molecules, atom sizes C =1.54 Å and N=1.5 Å should be considered.

Microscopic images were obtained using an HRTEM TITAN 60-300 instrument with an X-FEG-type emission gun operating at 80 kV. AFM imaging was performed with a Multimode 8 microscope under the control of a Nanoscope V controller (Bruker). The samples were prepared by drop-casting g-C_3N_4 or X/g-C_3N_4 suspension on the HOPG (highly ordered pyrolytic graphite) surface and then dried in air. AFM substrates were mounted on metallic discs using adhesive tape. Before sample deposition, a top layer of HOPG was peeled off using scotch tape to give a clean and atomically flat surface. All experiments were performed under ambient conditions at room temperature. Standard ScanAsyst-Air probes from Bruker were used.

Temperature programmed reduction runs (TPR, in 5% H_2 in He) were performed in a quartz tube reactor connected to a quadrupole mass spectrometer (HPR 60, Hiden). Typically 45 mg of the catalyst sample was used for each test. The sample was heated from RT to 773 K with a ramp rate of 10 K min^{-1}.

2.2. CO_2 hydrogenation tests

The CO_2 hydrogenation tests were performed under atmospheric pressure in a Catlab system (Hiden, UK), equipped with a tubular fixed-bed reactor (5 mm external diameter) and an online mass spectrometer (MS Hiden, HPR 60). Usually, 30 mg of the sieved catalyst (20-40 mesh) was loaded into the reactor, and the reactor was purged with He for ca 30 min at room temperature (RT). The temperature was measured using two thermocouples to increase accuracy. Before testing, the catalysts were pre-reduced *in situ* upon heating from RT to 773 K at a constant rate of 10 K min^{-1} under a flow of 5% vol.

H_2/He. After reaching 773 K, the temperature was maintained constant for 3h, and then the sample was cooled to RT in He flow. A total gas flow rate of 70 ml min^{-1} was kept within all experiments (GHSV = 5.1 $\times$ 10^3 h^{-1}, at 1 atm and 293 K). After that, the feed was switched to CO_2/H_2 in the molar ratio 5/1 in He balance. The pre-mixing fluxes of high-purity gases (CO_2, H_2, He) independently calibrated via Bronkhorst MFCs were used. Reaction tests were run in the temperature-programmed mode by heating up the reactor from RT to 773 K with a rate of 10 K min^{-1}. A real-time gas analyzer (MS Hiden, HPR 60) was used to analyze exhaust gas. MS was set to the MID mode, and following different m/z signals of CO, CO_2, H_2O, O_2, CH_4, CH3OH, HCHO, HCOOH, and H_2 were monitored continuously by quadrupole detector, with cross-sensitivity software corrections, neglecting the eventual formation of traces of C_{2+} hydrocarbons. Equilibrium calculations of the methanation process at 1 bar were performed using CEA software (NASA).

2.3. Catalyst toxicity tests

2.3.1 Cell line

Spontaneously transformed aneuploid immortal keratinocyte cell line from histopathologically normal adult human skin (HaCaT) were obtained from CLS (Cell Lines Service GmbH, Eppelheim Germany). The HaCaT cells were cultured in DMEM/F12 (1:1 mixture of DMEM and Ham's F-12) medium supplemented with 4.5 g/L glucose, 2 mM L-glutamine (Corning Inc., USA), 10% fetal bovine serum (FBS, Gibco, BRL, UK), and 100 U/mL penicillin and 100 μg/mL streptomycin (Sigma Chemical Co.). HaCaT cells were cultured in an incubator at 37 °C in 95% air and 5% CO_2 humidified atmosphere. The medium was changed every 2-3 days. The cells were rinsed with the use of a Dulbecco's Phosphate Buffered Saline (DPBS) without Ca^{2+}/Mg^{2+} (Corning, Co.) and harvested with 0.25% Trypsin-EDTA (Sigma Aldrich).

2.3.2 Assessment of catalysts cytotoxicity

g-C_3N_4 and X/g-C_3N_4 (X=Ni, Cu, CuNi) catalysts powders were suspended in serum-free culture DMEM/F12 medium as a 1 mg/mL stock solution and dispersed for 30 min using a bath sonicator (Ultron U-509, Poland) at room temperature. Then, the stock solution was serially diluted to reach the required catalysts colloid concentrations (ranging from 3.125 μg/mL to 500 μg/mL) and further added to the cells for subsequent experiments.

The HaCaT cells at a density of 2x10^4 cells per well were seeded on 96-well plates (Corning Inc., USA) and allowed to attach overnight. The next day, the culture medium was removed, the cells were DPBS rinsed and treated with various concentrations of NanoParticles powders (3.125 - 500 µg/mL) suspended in a serum-free medium (SFM), 200 µL/well of total volume, or in the case of control cells in SFM alone. Cells viability/proliferation was measured after 24 h by the application of the colorimetric MTS assay, in which yellow tetrazolium salt (MTS, 3-(4,5-dimethylthiazol-2-yl)-5-(3-carboxymethoxyphenyl)-2-(4-sulfophenyl)-2H-tetrazolium) is usually converted by NAD(P)H-dependent dehydrogenase enzymes in viable and metabolically active cells to generate a colored formazan dye that is soluble in cell culture media. The cells were incubated at 37 ^{o}C for 3 h with the MTS reagent (20 µL/well, CellTiter 96® AQueous One Solution Cell Proliferation Assay, Promega Co.). The quantitative analysis of product was performed by the measurement of absorbance at λ=490 nm with the use of a plate reader (Synergy H1 Microplate Reader, BioTek Instruments, Inc., Winooski, VT, USA) and Gen5 Microplate Reader and Imager Software (BioTek) All experiments were performed in three biological repeats. Cell viability was compared with the control cells.

2.3.3 Evaluation of cell morphology

The HaCaT cells were seeded on 6-well plates (Corning Inc., USA) at a density of 4x10^4 cells per well and left overnight in the incubator to adhere. The next day, the culture medium was removed, the cells were DPBS rinsed and treated with various concentrations of catalysts (3.125-500 µg/mL) in a new culture medium without FBS (SFM) or cultured in the SFM alone (control cells). The cells were evaluated in an optical microscope (Olympus Optical co. BX51). The images were captured and processed by ScopeImage 9.0 software.

2.3.4 Statistical analysis

Statistical analysis for MTS assay was performed by one-way analysis of variance (ANOVA) with Tukey's post hoc test in the Statistica 12 software (StatSoft Inc., USA). The data is shown as the mean ± standard deviation (SD) (*$p < 0.05$, **$p < 0.01$, ***$p < 0.001$; n=18 per concentration from three independent experiments). Plots were created using GraphPad Prism *v8*.2.1 (*GraphPad* Software Inc., San Diego, USA).

References

1. I. S.Pieta, A. Rathi, P. Pieta, R. Nowakowski, M. Holdynski, M. Pisarek, A. Kaminska, M. B. Gawande and R. Zboril, *Appl. Catal. B-Environmental*, 2019, **244**, 272-283.
2. I. S.Pieta, *Patent, P-412277*, 2015.
3. I. S.Pieta, P. Pieta and R. Nowakowski, *Patent, P.434122* 2020.
